# The Pure Food Bill

**Published:** 1906-07

**Authors:** 


### THE PURE FOOD BILL.

The Pure Food Bill, after seventeen years before Congress, finally passed the House June 24th. It had already passed the Senate.

The bill as agreed upon makes it a misdemeanor for any person to manufacture, sell or offer for sale any article of food, drugs, medicines or liquors which is adulterated or mis-branded or which contains any poisonous substances.

Section 2 prohibits the introduction into any State or Territory or the District of Columbia from any State, Territory or district or any foreign country, or the shipment to any foreign country, of any food adulterated or misbranded within the meaning of the act under a penalty of not exceeding a fine of $200 for the first offense and $300 or one year's imprisonment, or both, for each subsequent offense.

Section 3 provides for rules and regulations for carrying out the act.

Section 4 provides for examination of specimens of food and drugs manufactured.

The standards for drugs are those recognized in the United States Pharmacopeia or national formulary. Confectionery is held adulterated if it contains any ingredient or coloring matter deleterious or detrimental to health. Foods are held adulterated if containing any substance reducing, lowering or injuriously affecting its quality or strength.

The use of preservatives by external application is permitted when the directions for the removal of such preservatives are printed on the covering of the package. Food products are declared adulterated if composed in whole or in part of a filthy, decomposed or putrid animal or vegetable substance or of any portion of an animal unfit for food, whether manufactured or not, or if the product of a diseased animal or one that has died otherwise than by slaughter.

The term misbranded applies to foods or drugs whose package or label bears any statement, design or device false or misleading, or falsely branded as to place where manufactured. Any article, however, which does not contain any poisonous ingredients shall not be deemed adulterated or misbranded when known under its distinctive name, or if compounds, imitations or brands are plainly indicated.

The term "brands" is construed to mean a mixture of like substances and does not exclude harmless coloring or flavoring ingredients.

Proprietors or manufacturers of foods which are not unwholesome are not required to disclose their formula.

Dealers are protected against prosecution if they hold the guaranty of the concern from whom they are purchased. The act is to take effect January 1 next.

